# Comparison of self-measured diurnal intraocular pressure profiles using rebound tonometry between primary angle closure glaucoma and primary open angle glaucoma patients

**DOI:** 10.1371/journal.pone.0173905

**Published:** 2017-03-23

**Authors:** Shaoying Tan, Nafees Baig, Linda Hansapinyo, Vishal Jhanji, Shihui Wei, Clement C. Tham

**Affiliations:** 1 Department of Ophthalmology and Visual Sciences, The Chinese University of Hong Kong, Kowloon, Hong Kong; 2 Department of Ophthalmology, Chinese PLA General Hospital, Beijing, China; 3 Hong Kong Eye Hospital, Kowloon, Hong Kong; 4 Department of Ophthalmology, Faculty of Medicine, Chiang Mai University, Chiang Mai, Thailand; 5 Department of Ophthalmology and Visual Sciences, Prince of Wales Hospital, Shatin, Hong Kong; Bascom Palmer Eye Institute, UNITED STATES

## Abstract

**Purpose:**

To document the diurnal intraocular pressure (IOP) profile with rebound tonometry performed by primary glaucoma patients in non-clinic environment.

**Patients and methods:**

Fifty-three medically-treated eyes of 31 primary angle closure glaucoma (PACG) and 22 primary open angle glaucoma (POAG) patients with no previous eye surgery were recruited. Diurnal IOP was measured 5 times per day at four-hourly intervals from 08:00 to 24:00 for 1 week in patients’ study eye using rebound tonometry in a non-clinic environment. The diurnal IOP profiles were compared between PACG and POAG eyes.

**Results:**

For both PACG and POAG eyes, mean patient-measured IOP was highest in the morning, gradually decreased over the course of a day, and was lowest by midnight (*p* < 0.001). The diurnal IOP fluctuation ± 1 standard deviation (SD), as documented by SD in daily IOP values, was lower in PACG group (1.6 ± 1.1 mmHg) than in POAG group (2.0 ± 1.2 mmHg; *p* = 0.049). The mean trough IOP ± 1 SD was higher in PACG group (12.9 ± 2.8 mmHg), compared to POAG group (11.5 ± 3.8 mmHg; *p* = 0.041). The mean IOP level at midnight ± 1 SD in PACG group (14.0 ± 3.2 mmHg) was higher than that in POAG group (12.1 ± 3.7 mmHg; *p* = 0.013).

**Conclusions:**

IOP in primary glaucoma patients was highest in the morning, and decreased over the course of a day in non-clinic environment. Treated diurnal IOP fluctuation seemed to be greater in POAG than PACG eyes.

## Introduction

Glaucoma is the second leading cause of irreversible visual impairment and blindness worldwide.[1] More than 60 million people in the world were affected by primary glaucoma in the year 2000.[1] According to angle status, primary glaucoma can be divided into primary open angle glaucoma (POAG) and primary angle closure glaucoma (PACG). For both types of primary glaucoma, intraocular pressure (IOP) elevation is a major risk factor for the onset and progression of glaucomatous optic neuropathy.[2–5] IOP reduction is the only proven clinical therapy to slow down glaucoma progression.[4]

IOP has been shown to fluctuate over time.[6] Recent evidence suggested that large diurnal IOP fluctuation may be an independent risk factor for glaucoma progression, in addition to the elevated IOP level.[7,8] A single measurement in the clinic might underestimate the real peak IOP in the non-clinic environment, especially in PACG where the IOP may be affected by a number of factors, such as the state of dilatation of the pupil, which in turn may be affected by ambient lighting. Unfortunately, the diurnal IOP profile of PACG eyes, especially in the more variable non-clinic environment, has yet to be documented. There has also been no comparison of diurnal IOP fluctuation of PACG to POAG eyes.

For accurate IOP measurement, Goldmann applanation tonometry (GAT) is the gold standard in clinical practice.[9] GAT has to be performed with topical anesthesia by trained healthcare professionals in a clinic setting. There are, therefore, inherent limitations with GAT measurements, such as the measurement environment and the time of the day of measurements. Rebound tonometry (RBT) allows easy and safe IOP measurement by paramedical personnel in the community.[10] It can also be used in children and non-cooperative patients.[11] Unlike applanation tonometry, RBT does not require the use of topical anesthesia nor fluorescence staining during IOP measurement,[12] and is less influenced by corneal properties.[13] The iCare ONE^®^ tonometer (iCare Finland, Oy, Finland) is one of the newer generation of RBT devices that allows glaucoma patients, after adequate training, to measure their own IOP safely and accurately outside the clinic environment.[11] Good correlation and agreement between rebound tonometry by patients and GAT by healthcare professionals had been evaluated in the published literature.[11,14–18] These allow diurnal IOP measurement at different times of the day and in ambient environments that might not be easily achievable in the past. To our knowledge, diurnal IOP in primary glaucoma patients, measured by the patients themselves using RBT in their ambient environments, has not been reported in the published literature.

In this study, the diurnal IOPs were self-measured by PACG and POAG patients using the RBT device (iCare ONE tonometer) in their ambient environments. The diurnal IOP profiles were compared between PACG and POAG eyes.

## Materials and methods

### Study subjects and ophthalmic examinations

Patients with PACG or POAG were prospectively recruited at the eye clinics of the Chinese University of Hong Kong and Hong Kong Eye Hospital between April 2012 and December 2013. Written informed consents were obtained from all study subjects. The study protocol and consent procedure was approved by the Ethics Committee for Human Research at the Chinese University of Hong Kong. The study adhered to the tenets of the Declaration of Helsinki. Authors do not have access to information that could identify individual participants during or after data collection.

Clinic IOP was measured by Goldmann applanation tonometry on a slit-lamp biomicroscope. The reading in mm Hg was rounded to the next higher integer. Two IOP measurements were taken but if the readings differed by greater than 2 mmHg, a third measurement would be taken. Clinic IOP was defined as the mean of two IOP measurements, or the median of three measurements. Angle status was determined with darkroom gonioscopy. Shaffer system was used for the grading of angle width. Grade 0 was defined as closed, and indentation will distinguish appositional from synechial angle closure. The diagnosis of glaucoma was based on characteristic glaucomatous optic nerve head morphology, and confirmed by Humphrey automated perimetry (Humphrey Field Analyzer II, Carl Zeiss Meditec, California, USA; Central 24–2 threshold test, size III white stimulus, with the foveal threshold test turned on). The criteria for glaucomatous visual field defect were adopted from a published report.[19] All eyes with secondary causes of ocular hypertension or glaucoma, and eyes with previous ocular surgery (except laser peripheral iridotomy and laser iridoplasty for PACG) were excluded. Best-corrected LogMAR visual acuity and ultrasound corneal pachymetry were performed on all study eyes.

We randomly selected one eye of a patient using a random number table if the patient had bilateral disease. If the patient had unilateral glaucomatous optic neuropathy, the diseased eye would be included. The patients received topical IOP-lowering eye drops, as indicated clinically.

### Patient-measured diurnal IOP in ambient environment

Before using the iCare ONE tonometer, all recruited patients participated in a 2-hour training session on the correct method for self-measurement of IOP using rebound tonometry. The training included watching a standard training video (http://www.youtube.com/watch?v=9Ov4VZXAZN4) with commentary by a study investigator who is experienced in using the self-rebound tonometry. Subjects also had hands-on training in using the tonometer under guidance and supervision. By the end of the training session, all subjects were assessed for their performance of the self-rebound tonometry to ensure that they followed a standard operating procedure. A hard copy of Icare ONE Quick Guide was distributed to every patient after the training session. All subjects were clearly instructed to remain in their usual living environment, e.g. at home, office or school, during the 1-week IOP documentation. All subjects were instructed to continue their usual activities of daily living, and to stop such activities only when they had to measure their IOP. Subjects were taught to measure their IOP in the upright sitting position, and eye drops were taken after the IOP measurements. The iCare ONE tonometer automatically acquires a mean IOP reading with every 6 consecutive automated contacts with the central cornea. Five IOP measurements were conducted every day at four-hourly intervals, from 08:00 to 24:00 (i.e. at 08:00, 12:00, 16:00, 20:00, and 24:00) for a week. At least three valid IOP readings were obtained at each time point. The readings and times of IOP measurements were automatically stored in the tonometer, and transferred to a computer for further analysis by the Icare^®^ LINK software. The median of all valid readings at each time point was used for statistical analyses. A new sterilized and disposable probe was used for each IOP measurement to avoid damaged probes affecting accuracy of measurement, and cross infection.

### Statistical analyses

Based on previous sample size calculation, we aimed to recruit 16 patients in the two groups. Subject would be excluded from the database if found missing data. The demographic characteristics of the study subjects were collected. The IOP value at each time point was calculated by averaging (mean) the IOPs measured at the same time point of every day during the whole study week. The diurnal IOP values at the 5 time points of the day constituted the ‘individual diurnal IOP profile’. The ‘group diurnal IOP profiles’ were acquired by the medians of the IOP values at the study time points of all recruited subjects within the group. Differences were compared by independent t-test (normal distribution) or Mann-Whitney U test (non-normal distribution). Normality of the data was tested by Shapiro-Wilk test. Categorical data were compared using the Pearson Chi-Square test or Fisher’s test. IOP differences among 5 time points were tested by Friedman test, and post-hoc test was further evaluated by Wilcoxon Signed Ranks test. A *p* < 0.05 was considered as statistically significant. The statistical significance in Wilcoxon Signed Ranks test was adjusted by Bonferonni’s corrections (*p* < 0.05/10 = 0.005). All statistical analyses were performed using SPSS software (version 20.0; SPSS Inc., Chicago, IL, USA).

## Results

### Demographics of the study subjects

Fifty-three patients with primary glaucoma (31 PACG and 22 POAG; Table 1) were recruited. Eleven of the 31 PACG eyes (35.5%) had history of acute angle closure (AAC). Thirty PACG eyes (96.8%) had received laser peripheral iridotomy (PI) previously. The one PACG eyes without PI were scheduled to receive PI after the study week. The mean spherical equivalent ± 1 SD was +1.05 ± 2.30 diopters (range, -5.25 to +5.50 diopters) in PACG group, and -5.75 ± 5.30 diopters (range, -17.00 to +2.25 diopters) in POAG group (*p* < 0.001). The mean vertical cup-to-disc ratio ± 1 SD was 0.64 ± 0.20 (range, 0.30 to 0.90) in PACG, and 0.79 ± 0.24 (range, 0.10 to 1.00) in POAG (*p* = 0.003). The mean ± 1 SD, and median (25^th^ percentile, 75^th^ percentile) extent of synechial angle closure in PACG group was 262.7 ± 126.5 degrees, and 325.0 (191.3, 360.0) degrees, respectively. The corresponding values in POAG group were 2.7 ± 10.9 degrees, and 0 (0, 0) degree (*p* < 0.001). The average mean deviation (MD) and pattern standard deviation (PSD) ± 1 SD, on Humphrey automated perimetry were -10.41 ± 7.72 dB (range, -30.00 to -2.00 dB) and 4.55 ± 2.75 dB (range, 2.00 to 13.00 dB) in PACG group, and -14.69 ± 7.75 dB (range, -31.00 to -2.00 dB) and 8.45 ± 3.66 dB (range, 1.00 to 14.00 dB) in POAG group, respectively (MD: *p* = 0.070, PSD: *p* = 0.009) (Table 1).

**Table 1:** Demographics of the study subjects.

	PACG (n = 31)	POAG (n = 22)	*p*
**Age, year**	62.4 ± 11.1 (41.2–86.2)	57.6 ± 11.1 (34.7–72.2)	0.065^
**Gender (Male/Female)**	10/21	18/4	0.001#
**Study eye (Right/Left)**	15/16	11/11	1.000#
**Previous AAC (Yes/No)**	11/20	0/22	0.001#
**Cataract (Yes/No)**	23/8	8/14	0.008#
**Number of drugs**	1.8 ± 1.6 (0.0–5.0)	2.5 ± 1.4 (0.0–5.0)	0.358^
**Spherical equivalent, Diopter**	1.05 ± 2.3 (-5.25–5.50)	-5.75 ± 5.30 (-17.00–2.25)	<0.001^
**BCVA, Snellen**	-0.22 ± 0.31 (-1.00–0.30)	-0.19 ± 0.30 (-1.00–0.15)	0.312^
**CCT, μm**	537.2 ± 31.3 (473.0–602.0)	546.1 ± 32.8 (500.0–632.0)	0.420^
**C/D Ratio**	0.64 ± 0.20 (0.30–0.90)	0.79 ± 0.24 (0.10–1.00)	0.003^
**Degree of Angle Closed, °**	262.7 ± 126.5 (0.0–360.0)	2.7 ± 10.9 (0.0–45.0)	<0.001^
**VF MD, dB**	-10.41 ± 7.72 (-30.00 –-2.00)	-14.69 ± 7.75 (-31.00 –-2.00)	0.070^
**VF PSD, dB**	4.55 ± 2.75 (2.00–13.00)	8.45 ± 3.66 (1.00–14.00)	0.009^

Data was presented as mean ± 1 SD (minimum–maximum). POAG: Primary Open Angle Glaucoma; PACG: Primary Angle Closure Glaucoma; AAC: Acute Angle Closure; PI: Peripheral Iridotomy; BCVA: Best-corrected Visual Acuity; CCT: Central Corneal Thickness; C/D Ratio: Vertical Cup-to-disc Ratio; VF: Visual Field; MD: Mean deviation; PSD: Pattern standard deviation; IOP: Intraocular Pressure; n: Number of subjects; SD: Standard deviation; CI: Confidence interval.

^ Mann-Whitney U test

^#^ Fisher’s Exact test

Overall, the mean number ± 1 SD of IOP-lowering drugs received by PACG eyes was 1.8 ± 1.6 (range, 0 to 5), whereas that of POAG eyes was 2.5 ± 1.4 (range, 0 to 5) (*p* = 0.358). Forty-four patients (25 PACG and 19 POAG) (83.0%) completed all 5 measurements each day in the study, while 9 patients (6 PACG and 3 POAG) (17.0%) had missed some time points. The percentage of IOP measurements missed in the 9 study patients ranged from 11.4% to 31.4%. Data from these 9 patients were included in the calculation of mean diurnal IOP and IOP fluctuation, and were therefore included in the diurnal IOP profile of the two groups. Data from these 9 patients were not included in the calculation of peak and trough IOP frequencies, because of their incomplete measurements during the study week.

### Description of diurnal IOP in PACG and POAG groups

In order to study the characteristics of diurnal IOP profile in primary glaucoma patients, the average IOP, peak IOP, trough IOP, and IOP fluctuation, were calculated in both PACG and POAG groups (Table 2). The difference in trough IOP was statistically significant between PACG and POAG groups (PACG: 12.9 ± 2.8 mmHg; range: 5.8–19.3 mmHg; POAG: 11.5 ± 3.8 mmHg; range: 5.9–23.1 mmHg; *p* = 0.041).

**Table 2:** Diurnal intraocular pressure fluctuation in primary angle closure glaucoma and primary open angle glaucoma groups.

	Total (n = 53)	PACG (n = 31)	POAG (n = 22)	*p* ^
**Average Diurnal IOP**	14.4 ± 3.9 (14.2 (11.2, 16.4), 7.1–28.4)	14.8 ± 3.6 (15.1 (12.9, 16.6), 7.1–26.7)	13.9 ± 4.3 (13.8 (10.7, 14.9), 9.1–28.4)	0.139
**Peak Diurnal IOP**	16.6 ± 4.9 (16.0 (13.1, 19.1), 7.8–34.9)	16.8 ± 4.6 (16.4 (13.3, 18.2), 7.8–31.6)	16.4 ± 5.4 (14.8 (11.9, 17.0), 10.9–34.9)	0.401
**Trough Diurnal IOP**	12.3 ± 3.3 (12.3 (9.7, 14.1), 5.8–23.1)	12.9 ± 2.8 (14.1 (11.6, 15.8), 5.8–19.3)	11.5 ± 3.8 (11.7 (9.7, 13.1), 5.9–23.1)	0.041
**Diurnal IOP fluctuation**				
** 1 SD of IOP values**	1.7 ± 1.2 (1.4 (1.1, 2.0), 0.3–6.3)	1.6 ± 1.1 (1.3 (0.9, 1.8), 0.3–5.1)	2.0 ± 1.2 (1.9 (1.3, 2.1), 0.8–6.3)	0.049
** Range of IOP values**	4.3 ± 2.8 (3.6 (2.6, 4.8), 0.6–13.5)	3.9 ± 2.7 (2.3 (1.6, 2.4), 0.6–12.4)	4.8 ± 2.9 (3.2 (2.2, 4.0), 1.6–13.5)	0.059
**IOP at each measured time point**				
** IOP at 08:00**	15.9 ± 5.1 (15.4 (12.5, 18.2), 7.4–34.9)	16.1 ± 4.8 (16.4 (12.5, 18.2), 7.4–31.3)	15.8 ± 5.7 (14.8 (11.9, 17.0), 9.4–34.9)	0.427
** IOP at 12:00**	14.7 ± 4.1 (14.1 (11.7, 16.4), 7.8–31.6)	15.0 ± 4.1 (14.6 (13.3, 16.4), 7.8–31.6)	14.3 ± 4.1 (13.6 (10.7, 16.0), 9.6–27.0)	0.283
** IOP at 16:00**	14.4 ± 4.0 (14.0 (11.9, 16.1), 7.3–29.1)	14.8 ± 3.5 (14.1 (12.8, 16.3), 7.6–26.3)	13.7 ± 4.5 (13.0 (10.8, 15.5), 7.3–29.1)	0.108
** IOP at 20:00**	13.9 ± 4.1 (14.1 (10.8, 16.0), 7.0–28.0)	14.3 ± 3.7 (14.7 (11.7, 15.8), 7.0–24.8)	13.4 ± 4.7 (12.8 (9.7, 15.9), 7.3–28.0)	0.259
** IOP at 24:00**	13.2 ± 3.5 (13.0 (10.5, 15.7), 5.8–23.1)	14.0 ± 3.2 (14.4 (11.6, 16.4), 5.8–19.3)	12.1 ± 3.7 (11.7 (9.7, 13.1), 5.9–23.1)	0.013

Data was presented as mean ± 1 SD, mmHg (median, mmHg (25^th^ percentile, mmHg, 75^th^ percentile, mmHg), minimum–maximum, mmHg). PACG: Primary angle closure glaucoma; POAG: Primary open angle glaucoma; IOP: Intraocular pressure; SD: Standard deviation; n: Number of subjects.

^ Mann-Whitney U Test

The mean ± 1 SD (median (25^th^ percentile, 75^th^ percentile; range) of IOP fluctuation (1SD of diurnal IOP values) was 1.6 ± 1.1 mmHg (1.3 (0.9, 1.8) mmHg; 0.3 to 5.1 mmHg) in PACG group, and 2.0 ± 1.2 mmHg (1.9 (1.3, 2.1) mmHg; 0.8 to 6.3 mmHg) in POAG group (*p* = 0.049) (Table 2). The POAG group therefore has a greater spread of IOP values over the course of a day than the PACG group.

### Diurnal IOP profiles in PACG and POAG groups

**Fig 1:**
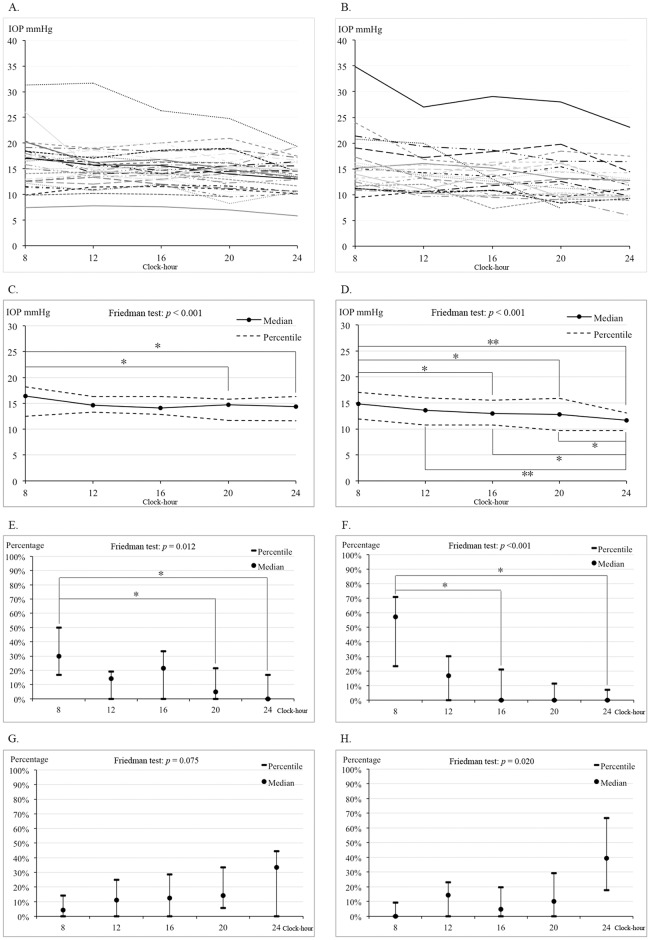
Diurnal IOP fluctuation in PACG and POAG patients. **(A)** The mean diurnal IOP profiles of 31 PACG patients. **(B)** The mean diurnal IOP profiles of 22 POAG patients. **(C)** The diurnal IOP profile of PACG group. The IOP at 08:00 was significantly higher than that at 20:00 (*p* = 0.001) and 24:00 (*p* = 0.001). **(D)** The diurnal IOP profile of POAG group. The IOP at 08:00 was significantly higher than that at 16:00 (*p* = 0.004), 20:00 (*p* = 0.004) and 24:00 (*p* < 0.001) and the IOP at 24:00 was significantly lower than 12:00 (*p* < 0.001), 16:00 (*p* = 0.004) and 20:00 (*p* = 0.002). **(E)** The frequency of peak diurnal IOP measured in PACG patients. The measurement at 8:00 was higher than that at 20:00 (*p* = 0.003) and 24:00 (*p* = 0.001). **(F)** The frequency of peak diurnal IOP measured in POAG patients. The measurement at 08:00 was higher than that at 16:00 (*p* = 0.003) and 24:00 (*p* = 0.001). **(G)** The frequency of trough diurnal IOP counted in PACG patients. **(H)** The frequency of trough diurnal IOP counted in POAG patients.

Fig 1A and 1B showed the diurnal IOP profiles of 31 PACG and 22 POAG patients, respectively. The mean IOP for both PACG and POAG groups during the study week was highest in the morning, relatively stable in daytime, and slowly decreased in the evening. Fig 1C and 1D showed the median, 25^th^ and 75^th^ percentiles of diurnal IOP profiles for PACG and POAG groups, respectively. In PACG group, the median IOP was 16.4 (12.5, 18.2) mmHg, 14.6 (13.3, 16.4) mmHg, 14.1 (12.8, 16.3) mmHg, 14.7 (11.7, 15.8) mmHg and 14.4 (11.6, 16.4) mmHg at 08:00, 12:00, 16:00 20:00 and 24:00, respectively (Table 2 and Fig 1C). There were statistically signfiicant differences between the 5 time points (Friedman test; *p* < 0.001). Wilcoxon Signed Ranks test further showed that the IOP at 08:00 was significantly higher than that at 20:00 (*p* = 0.001) and 24:00 (*p* = 0.001) in PACG eyes (Fig 1C). In POAG group, the median IOP was 14.8 (11.9, 17.0) mmHg, 13.6 (10.7, 16.0) mmHg, 13.0 (10.8, 15.5) mmHg, 12.8 (9.7, 15.9) mmHg, and 11.7 (9.7, 13.1) mmHg for the 5 measurement time points, respectively (Table 2 and Fig 1D). The diurnal IOP in POAG eyes were significantly different among the 5 time points (*p* < 0.001). The IOP at 08:00 was significantly higher than that at 16:00 (*p* = 0.004), 20:00 (*p* = 0.004) and 24:00 (*p* < 0.001). In addition, IOP at 24:00 was significantly lower than other time points (12:00 (*p* < 0.001), 16:00 (*p* = 0.004) and 20:00 (*p* = 0.002)) in POAG group (Fig 1D).

There was also statistically significant difference in mean IOP at midnight (24:00) between the PACG group (14.0 ± 3.2 mmHg) and the POAG group (12.1 ± 3.7 mmHg) (*p* = 0.013).

### Frequency of peak and trough IOP at study time points

The frequencies of peak and trough IOP at study time points were counted in both PACG and POAG patients who completed all 5 measurement time points for all 7 days (25 PACG and 19 POAG patients). The percentage of occurrence throughout the course of a day is presented in Table 3. In PACG group, the peak IOP was most often noted at 08:00 (median = 30.0%, 25^th^ percentile = 16.7%, 75^th^ percentile = 50.0%), followed by 16:00 (median = 21.4%, 25^th^ percentile = 0%, 75^th^ percentile = 33.3%). The percentage of occurrence of peak IOP was significantly different among the 5 time points (*p* = 0.012), the occurrence at 08:00 was higher than that at 20:00 (*p* = 0.003) and 24:00 (*p* = 0.001; Fig 1E). In contrast, trough IOP was most frequently documented at 24:00 (median = 33.3%, 25^th^ percentile = 0%, 75^th^ percentile = 44.4%), and least frequently documented at 08:00 (median = 0%, 25^th^ percentile = 0%, 75^th^ percentile = 14.3%). However, the frequencies of trough IOP did not show any statistically significant differences among the 5 time points (Fig 1G).

**Table 3:** Percentage of peak and trough diurnal intraocular pressure occurrence in primary angle closure glaucoma and primary open angle glaucoma groups.

	Time	Percentage of occurrence		*p*-value^
		PACG (n = 25)	POAG (n = 19)	
**Peak**	**08:00**	30.0 (16.7, 50.0)	57.1 (23.2, 70.8)	0.230
	**12:00**	14.3 (0, 19.0)	16.7 (0, 30.3)	0.492
	**16:00**	21.4 (0, 33.3)	0 (0, 21.1)	0.108
	**20:00**	4.8 (0, 21.4)	0 (0, 11.3)	0.486
	**24:00**	0 (0, 16.7)	0 (0, 6.9)	0.283
**Trough**	**08:00**	4.2 (0, 14.3)	0 (0, 9.2)	0.514
	**12:00**	11.1 (0, 25.0)	14.3 (0, 23.2)	0.961
	**16:00**	12.5 (0, 28.6)	4.8 (0, 19.6)	0.360
	**20:00**	14.3 (5.6, 33.3)	10.0 (0, 29.2)	0.380
	**24:00**	33.3 (0, 44.4)	39.3 (17.9, 66.7)	0.237

Data was presented as median, % (25^th^ percentile, %, 75^th^ percentile, %). PACG: Primary angle closure glaucoma; POAG: Primary open angle glaucoma; n: Number of subjects.

^ Mann-Whitney U Test

A similar situation was found in the POAG group. Peak IOP was most frequently documented at 08:00 (median = 57.1%, 25^th^ percentile = 23.2%, 75^th^ percentile = 70.8%). The percentage of peak IOP occurring among the 5 time points also showed a statistically significant difference (*p* < 0.001), the frequency at 08:00 being significantly higher than those at 16:00 (*p* = 0.003) and 24:00 (*p* = 0.001; Fig 1F). The trough IOP in the POAG group was most frequently documented at 24:00 (median = 39.3%, 25^th^ percentile = 17.9%, 25^th^ percentile = 66.7%) and least frequently at 08:00 (median = 0%, 25^th^ percentile = 0%, 75^th^ percentile = 9.2%). Statistically significant difference was found among the 5 time points (*p* = 0.020); however, individual differences did not reach statistical significance after Bonferroni corrections (Fig 1H).

The peak IOP was most frequently documented at 08:00 in the morning and the trough IOP in the POAG group was most frequently documented at mid-night. However, there was no statistically significant difference in the frequencies of peak or trough IOP between PACG and POAG groups (Table 3).

## Discussion

Apart from mean IOP, IOP fluctuation may be another risk factor for the onset and progression of glaucoma.[6,8,20–22] A retrospective review showed that peak diurnal IOP was on average 4.9 mm Hg higher than the ‘maximum IOP’ documented routinely in clinic. More than half (51.7%) of the patients’ peak IOP occurred outside usual clinic hours.[23]

In the settings of many clinics, diurnal IOP profile is often obtained by arranging patient’s clinic visits, and therefore IOP measurements, at different times of the day for each patient subject. It can also be achieved by measurement of IOP at regular pre-determined intervals with the subjects admitted to a ward or a sleep laboratory.[8,24,25] The key advantage of such methodologies is that the IOP measurement by eye care professionals may be more accurate and consistent, but one important disadvantage is that the patient subjects are completely removed from their usual ambient environments and normal routines of daily living when the IOP measurements are made. This disadvantage of conventional methodology may be of special relevance in PACG, where the patients’ ambient environment, e.g. the level of illumination, and daily activities, might theoretically affect the IOP level through various mechanisms, such as the dilatory status of the pupil.[26–29] The illumination level in clinic is usually standard and consistent, and may therefore not simulate patients’ usual living environments. Previous studies have employed rebound tonometry to document diurnal IOP in children in the home environment. The rebound tonometry in this previous study was performed by carers on children, and the IOP measurements were conducted over only one to two days.[16] In our study, the latest rebound tonometer (iCare ONE tonometer, iCare Finland, Oy,. Finland) was used, which allowed the trained study subjects to measure their own IOP in their home or work environment, and thus allowing the diurnal IOP measurements to be conducted over the longer duration of 7 days. As there is a significant fluctuation in IOP, both in the short and medium term, measurements over a longer period of time are essential to obtain a more representative data set in each individual patient. To our knowledge, this is the first study that documented diurnal IOP profile in the patients’ ambient environments, without removing the subjects from their usual activities of daily living, continuously for 1 week.

Previous studies have documented the diurnal IOP fluctuation in POAG and PACG eyes,[5] and also in primary angle closure suspects (PACS) and primary angle closure (PAC).[30] Our findings on diurnal IOP pattern were similar to previous studies in POAG patients, with the highest mean IOP at 08:00, which then decreased gradually during the course of the day, until the lowest mean IOP recorded at midnight.[16,17,30,31] The diurnal IOP pattern in PACG eyes had a similar trend to the pattern in POAG in this present study. This finding is different from the another previous study that showed the peak IOP tended to occur more frequently in the afternoon in PACG eye with prior iridotomies.[5] In this study, the mean IOP at 08:00 was significantly higher than the mean IOP at midnight (00:00) in both PACG (*p* < 0.001) and POAG (*p* < 0.001) eyes, and the peak IOP was most frequently documented at 08:00 in both PACG and POAG groups. However, the range of diurnal IOP in our cohort was smaller, with a median IOP range of 2.3 mmHg in PACG and 3.2 mmHg in POAG, whereas previous studies showed a range of approximately 4 to 5 mmHg in glaucoma eyes.[30] The possible explanation for the different diurnal range of IOP fluctuation may be due to IOP measurements in the clinic setting versus the patients’ ambient environments. It may also be due to the fact that the enrolled subjects in the previous study did not yet have any medical or surgical treatment.

The mean IOP appeared to be higher in the POAG group than in the PACG group at all time points, but only the difference in mean IOP at midnight between the two groups reached statistical significance. In terms of IOP fluctuation parameters, the SD of diurnal IOP was lower and the trough IOP was higher in PACG eyes compared to POAG eyes. This might be explained by the differences of number and type of medicine between PACG and POAG patients taking. It also raised a possibility that a greater influence of light on the dilatation status of the pupil for PACG eyes as compared to POAG eyes.[28] Slower out-flow of the aqueous humor might reduce the reduction of IOP at nighttime in PACG eyes.[27] This may partly explain why the mean IOP in PACG eyes was higher than in POAG eyes at 24:00, and also why the overall IOP fluctuation was smaller in PACG eyes than in POAG eyes.[5,30] However, the role of angle status, medications and lighting conditions could not be concluded from the current study. Further studies maybe necessary to investigate the factors associated with IOP fluctuation in ambient environments.

The current study has a few limitations. First, the patients themselves conducted all of the IOP measurements. The accuracy of the IOP measurements depended on the training and skills of the patients. A total of 6 readings were collected for each IOP measurement. The rebound tonometer is able to recognize wrong positioning or large variations, and automatically delete possibly unreliable readings. Only the more consistent readings would be stored, allowing us to ensure a certain degree of compliance with the measurement protocol. In addition, we provided a 2-hour standard training program in using the rebound tonometer to all study patients to minimize the skill effect, with an evaluation at the end of training, to ensure accuracy and consistency of the IOP measurements. Three IOP readings were documented by both Goldmann applanation tonometry and self- conducted rebound tonometry on all of the participants after the training program. The within measurement intra-class correlation coefficient (ICC) of Goldmann applanation tonometry and rebound tonometry were 0.98 (95% confidence interval (CI): 0.97–0.99) and 0.94 (95% CI: 0.90–0.96), respectively. Although rebound tonometer was showed may underestimate the IOP compared to Goldmann applanation tonometry measurements in previous studies,[32] We evaluated the correlation and agreement between patient-measured IOP using rebound tonometry with ophthalmologist-measured IOP using Goldmann applanation tonometry in our cohort, and found that both modes of IOP measurements correlated well with each other, a good agreement was also identified with a mean difference ± 1 standard deviation (SD) of 0.15 ± 0.65 mmHg (*p* = 0.682) between Goldmann applanation tonometry and rebound tonometry. The between-measurement ICC of these two tonometries was 0.72.[33] Second, this study did not include normal subjects as controls. Previous studies showed that the diurnal IOP fluctuation was greater in glaucoma eyes, including POAG,[34–38] ocular hypertension[39] and PACG,[30] than normal eyes.[5] Further studies should be investigated on the diurnal IOP in glaucoma and normal eyes under same conditions. Third, all patients were under medical treatment to control IOP, whereas the PACG eyes had received prior laser iridotomy. The IOP profiles we recorded were therefore medically-treated IOP profiles, even though we excluded those eyes with prior surgical interventions that could significantly alter aqueous flow. To limit the drug effect, the participants were recommended to apply eye drops after self-measured IOP using rebound tonometry. Furthermore, as we found that the peak IOPs mostly occurred at 08:00, which was the first time point of the day to document IOP, the actual peak IOP might happen during the sleep time.[24] A study with continuous IOP monitoring (including measurement of nocturnal IOP during sleep), if possible, would provide very valuable data to increase our understanding of diurnal IOP patterns.

In summary, mean patient-measured IOP in the home environment was highest at 08:00, tended to drop over the course of a day, and was lowest by midnight, in both PACG and POAG eyes in this study. The PACG eyes had lower diurnal IOP fluctuation than POAG eyes, and higher midnight IOP. This study demonstrated the feasibility of diurnal IOP documentation by patients themselves using rebound tonometry in their ambient environments. Such measurements may potentially be of clinical relevance in patient management.

## Supporting information

S1 File Data. (XLSX)
